# Evacuation or Shelter in Place in Assisted Living: Adherence to Emergency Evacuation Orders for Hurricane Dorian

**DOI:** 10.1093/geroni/igaa057.2428

**Published:** 2020-12-16

**Authors:** Lindsay Peterson, Joseph June, Nazmus Sakib, Kathryn Hyer

**Affiliations:** 1 University of South Florida, Tampa, Florida, United States; 2 School of Aging Studies, University of South Florida, Tampa, Florida, United States

## Abstract

Hurricane Dorian devastated parts of the Bahamas in 2019 with 185-mph winds. As it moved toward Florida, the state went on alert. This paper examines whether assisted living communities (ALCs) in affected counties evacuated or sheltered in place in the context of emergency management communications concerning evacuation. In 16 coastal counties, 66 ALCs were under mandatory evacuation orders, but 12 sheltered in place. Of 603 ALCs not under orders, 17 evacuated. Seven ALCs evacuated contrary to orders in one county, which issued a mandatory order Sept. 1, delayed it to Sept. 2 as Dorian weakened, and lifted it Sept. 4. Interviews with ALC administrators and emergency managers will be used to understand more about their decisions. Given prior findings that evacuation (versus sheltering in place) increases the mortality and morbidity risk of long-term care (LTC) residents, this research suggests a need for clearer LTC evacuation standards and communication. Part of a symposium sponsored by Disasters and Older Adults Interest Group.

